# Simple Can Be Effective: A Brief Naloxone Training in Preclinical Medical Education

**DOI:** 10.7759/cureus.104867

**Published:** 2026-03-08

**Authors:** Joel P White, Lizabelle Martin, Claudia Morris, Alexandra Montefalcone, David Trotter, Elisabeth Conser

**Affiliations:** 1 School of Medicine, Texas Tech University Health Sciences Center, Lubbock, USA; 2 Department of Family Medicine, Texas Tech University Health Sciences Center, Lubbock, USA; 3 Department of Pediatrics, Texas Tech University Health Sciences Center, Lubbock, USA

**Keywords:** harm reduction education, naloxone, opioid-related disorders, overdose prevention, preclinical medical students, student-led initiatives

## Abstract

Background

Opioid overdose remains a leading cause of preventable mortality in the United States. Early education on overdose recognition and naloxone administration is recommended in medical training, yet its implementation in preclinical curricula is often limited by time and resource constraints. This study evaluated the efficacy of a brief, didactic naloxone training integrated into a mandatory preclinical wellness session in improving first-year medical students’ confidence in opioid knowledge, overdose recognition, and naloxone administration.

Methods

The first-year medical students at a single U.S. medical school were invited to complete anonymous pre- and post-training surveys surrounding a 12-minute naloxone training video. Survey items used a five-point Likert scale assessing confidence in opioid knowledge, overdose recognition, appropriate response actions, and understanding of naloxone. Pre- and post-survey responses were analyzed as independent samples using two-tailed Mann-Whitney U tests. Effect sizes were calculated using rank-biserial correlation.

Results

A total of 151 pre-survey and 133 post-survey responses were analyzed. Post-training responses demonstrated significantly higher confidence across all survey questions, including defining opioids, recognizing opioid overdose, knowing appropriate actions to take, and understanding naloxone (all p<0.001). Large effect sizes were observed for these outcomes (rank-biserial correlation range: 0.65-0.77). Participants also reported high confidence in their ability to administer intranasal naloxone following the intervention (median (IQR): 5 (4-5)).

Conclusions

A brief, resource-efficient naloxone training significantly improved preclinical medical students’ self-reported confidence in opioid overdose recognition and naloxone administration. This intervention demonstrates a scalable approach for integrating harm-reduction education into preclinical medical curricula and beyond.

## Introduction

In the United States, drug overdose remains one of the highest causes of death in adults, especially overdoses involving opioids and opioid analogs [1]. Per the Centers for Disease Control and Prevention (CDC), over 1000 daily visits to the emergency department are due to opioid toxicity, which claims 91 lives a day [1,2]. Opiates have been approved as an effective treatment for pain for over 70 years now, but their euphoric side effects cause them to be increasingly addictive [2]. With this continued epidemic, there has been an increase in programs that offer free over-the-counter naloxone to communities in addition to training on the recognition and interventions in case of an overdose [3,4]. Many programs have been implemented, but they each share a common goal: increasing awareness and recognition of opioid overdoses while providing knowledge on how to intervene.

The American Psychiatric Association guidelines state that there should be education on opioids and opioid use disorder in every stage of medical training [5]. In medical education, there has been an increased interest in establishing early education on opioid overdose identification and intervention [6,7]. Additionally, providing resources such as free naloxone to students has also been increasingly popular [8]. Current educational activities provided to preclinical medical students on opioid overdose and naloxone competencies as represented in the literature have been conducted through didactic sessions. These sessions are reported to be anywhere from one to two hours in length and include some element of simulation [6,9]. Each method was shown to be efficacious and their specific objectives varied. There are educational programs regarding opioid overdose and intervention represented in other study populations outside of preclinical medical students as well [10].

Based on descriptive statistics from a previous unpublished survey, our team found there was a need for early training in opioid overdose recognition and intervention among first-year medical students at our institution. In response, our team developed a short but novel training that can be integrated into the preclinical wellness curriculum at our institution. This study aimed to evaluate the efficacy of the developed training session in achieving the sought-after learning objectives. The training was designed to be simple, straightforward, and short while still providing adequate objectives and effective didactic teaching to address the lack of naloxone administration training in preclinical curriculum. This design was utilized to easily integrate training into the time-demanding preclinical curriculum. The training content was also created in relevance to the opioid epidemic in the geographic region, specifically to have additional relevance for students at our institution. By integrating this material into preclinical curriculum, our team addressed the need for early education while equipping future physician leaders to act in the face of an opioid overdose outside of the clinical setting.

The primary outcome of this study was to determine the efficacy in increasing confidence levels of preclinical medical students in subject areas surrounding opioids, naloxone, and overdose management. Secondarily, our team wanted to test the feasibility and scalability of integrating a simple training into the first-year preclinical curriculum while utilizing free local resources to distribute naloxone nasal spray to students.

## Materials and methods

Study design

This study was conducted as a single-institution, pre-post educational intervention using an anonymous survey assessment.

Participants

At a single U.S. medical school, a total of 182 first-year medical students attended a mandatory wellness lecture in March 2025. Participants were asked to complete a pre-survey, undergo training, and complete a post-survey upon conclusion of the mandatory wellness session. Although the previously scheduled wellness session was mandatory, completion of the materials provided by our team was optional. Participants received a free box of NARCAN® Nasal Spray (Emergent BioSolutions, Gaithersburg, MD, USA) upon conclusion of the session.

Ethics statement

This study was approved by the Quality Improvement Review Board (QIRB) as a program implementation and evaluation.

Narcan training initiative

Our team of four medical students and two medical doctorate supervisors developed a simple training video recording of a didactic lesson. The recording was played for participants following an in-person wellness lecture and lasted just under 12 minutes in duration. The team chose a wellness lecture over substance use disorders to complement the theme. Team members were present to address any questions or comments from participants. The video featured four students teaching through a recorded PowerPoint created to meet the learning objectives. The content of the PowerPoint video was developed using a review of the relevant literature and published regional data. It was refined with input from one supervising physician to ensure clinical accuracy and appropriateness for the target audience. The objectives of the educational material included the following: (1) define opioids through their mechanism and clinical uses, (2) explain opioid use disorder both clinically and epidemiologically, (3) describe the signs of an opioid overdose, (4) provide intervention protocol via NARCAN® (naloxone) along with mechanism of action, and (5) demonstrate the administration of NARCAN® (naloxone). Objectives 4 and 5 were designed and executed regarding intranasal naloxone only. This is due to the availability of intranasal naloxone and previous findings that participants are more likely to use this form of medication compared to intramuscular naloxone [11]. NARCAN® Nasal Spray was available for participants to take freely following the training.

Pre- and post-training surveys

A survey of nine questions was administered to participants before and after the training session was conducted. Seven out of the nine questions were identical on each survey and each question utilized an “I” statement: for example, “I am confident in my ability to define or explain what an opioid is”. Responses were recorded using a five-point Likert scale, where “1” indicated strong disagreement and “5” indicated strong agreement. The survey statements were developed by the study team for the purposes of this program evaluation and were not previously validated. The statements evaluated self-confidence level on the knowledge of opioids, naloxone, overdose recognition, and previous coursework or clinical experience on the topics. Content exclusive to the pre-survey assessed the comfort levels of participants’ theoretical naloxone administration abilities with and without training. Content exclusive to the post-survey directly assessed confidence and understanding of naloxone administration. Survey responses did not require any identification of participants. The term ‘NARCAN®’ was used in survey items because it reflects the labeling on the intranasal naloxone product displayed in the training and distributed to participants. All submitted surveys were complete, and no missing data was identified, allowing for the inclusion of all available responses. 

Analysis

Survey responses were recorded using Google Forms (Google LLC, Mountain View, CA, USA) immediately before and immediately after the training video in March 2025. Participants accessed the survey electronically through a personal device. The responses were analyzed by the first author.

Survey data was imported from a Microsoft Excel (Microsoft, Redmond, WA, USA) file and analyzed using Python (version 3.11.4; Python Software Foundation, Wilmington, DE, USA) with established statistical libraries. A total of 151 pre-survey responses and 133 post-survey responses were included in the analysis. Descriptive statistics were calculated for each survey question. A two-tailed Mann-Whitney U test was used to assess the differences in survey responses between pre- and post-survey groups. This nonparametric test was selected due to the ordinal nature of survey responses, the absence of assumptions regarding normality, and the independence of pre- and post-survey samples due to differing response rates and anonymity of participants [12]. The effect size for each comparison was calculated using the rank-biserial correlation, defined as 

$r_{rb} = 1 - \frac{2U}{n_{1} n_{2}}$

where is the Mann-Whitney U statistic and and are the sample sizes of the two groups. Rank-biserial correlation values of 0.10, 0.30, and 0.50 were interpreted as small, medium, and large effects, respectively. Statistical significance was defined as a p-value <0.05.

## Results

For the pre-survey, there was a response rate of 85.16% while the response rate for the post-survey was 73.06%. The pre-survey had unique questions that focused on comfort levels of participants in intervening in cases of overdose as seen in Table 1. The average response when considering acting without a prior special training was 2.23 with a standard deviation of 1.30 on the Likert scale. When considering acting only after having a special training, the average response was higher (mean: 3.63±1.12). The median (interquartile range) also differed at 2 (1-3) and 4 (3-4), respectively.

**Table 1:** Descriptive statistics for questions unique to the pre-survey. n: number; IQR: Interquartile Range; SD: standard deviation.

Question	n	Median (IQR)	Mean±SD
I would feel comfortable administering Narcan for an emergent opioid overdose even without special training.	151	2 (1-3)	2.23±1.30
				I would feel comfortable administering Narcan for an emergent opioid overdose even only after special training.	151	4 (3-4)	3.63±1.12

Questions included in both the pre- and post-survey assessed the efficacy of the training in meeting the objectives specified above. These questions center around self-identified confidence in the understanding of opioids, identifying an opioid overdose, knowing what meaningful actions to take in the setting of an overdose, and an understanding of NARCAN®. Other questions in both surveys assess previous experiences and coursework surrounding the subjects covered by the Narcan Training Initiative. For each question listed in Table 2, the means of the post-survey questions were higher compared to the pre-survey. Additionally, for the questions assessing confidence in defining opioids, recognizing overdose, knowing what action to take and defining NARCAN®, the standard deviation of the mean was lower for each from the pre-survey to the post-survey. The mean of the responses regarding prior experiences and coursework on the material are also higher in the post-survey compared to the pre-survey, while the standard deviations are higher. A similar trend for every question included in Table 2 is seen regarding the median (IQR) from pre-survey to post-survey responses. The Mann-Whitney U test statistic for each question showed significantly different median (IQR) responses from pre-survey to post-survey. The effect size for questions regarding defining opioids is greater than 0.50, while the effect size for questions on recognizing overdose, knowing what action to take, and defining NARCAN® are all greater than 0.70.

**Table 2:** Mann Whitney U Test comparison of questions included in both the pre- and post- survey. n: Number; IQR: interquartile range; SD: standard deviation; r_rb_: Rank-biserial correlation.

Question	Group	n	Mean±SD	Median (IQR)	p-value	r_rb_
I am confident in my ability to define or explain what an opioid is.	Pre	151	3.05±1.12	3 (2-4)	<0.001	0.65
	Post	133	4.35±0.63	4 (4-5)		
If I saw an opioid overdose, I would recognize it.	Pre	151	2.55±1.19	2 (2-3)	<0.001	0.76
	Post	133	4.32±0.68	4 (4-5)		
If I was present when someone overdosed on opioids, I would know what to do.	Pre	151	2.52±1.29	2 (1-3)	<0.001	0.77
	Post	133	4.49±0.64	5 (4-5)		
I am confident in my ability to define or explain what NARCAN® is and is used for.	Pre	151	2.89±1.22	3 (2-4)	<0.001	0.74
	Post	133	4.58±0.59	5 (4-5)		
I have seen first-hand the effects of opioid substance abuse.	Pre	151	2.11 ± 1.40	1 (1-3)	<0.001	0.28
	Post	133	2.95 ± 1.65	3 (1-5)		
I have had previous coursework or other training about opioid substance use.	Pre	151	2.28 ± 1.41	2 (1-3)	<0.001	0.27
	Post	133	3.08 ± 1.64	3 (1-5)		
I have had previous clinical experience that expanded my understanding of opioid substance use.	Pre	151	2.20 ± 1.36	2 (1-3)	0.0019	0.20
	Post	133	2.80 ± 1.64	3 (1-5)		

Questions unique to the post-survey (Table 3) centered on understanding and confidence in the appropriate actions to take in the event of an opioid overdose. The mean response on understanding how to administer NARCAN® Nasal Spray was 4.59±0.60. The mean response of participants feeling equipped to act in this situation was 4.55±0.61. The median (IQR) response was 5 (4-5) for each of the questions unique to the post-survey.

**Table 3:** Descriptive statistics of questions unique to the post-survey only. n: number; IQR: Interquartile Range; SD: standard deviation.

Question	n	Median (IQR)	Mean±SD
I understand how to administer Narcan in the event of an emergent opioid overdose.	133	5 (4-5)	4.59±0.60
				I feel equipped to administer Narcan for an emergent opioid overdose.	133	5 (4-5)	4.55±0.61

## Discussion

The overarching goal of this study was to identify the efficacy of a short intervention in content areas regarding opioids, naloxone, and opioid overdose identification and management. This intervention is novel in comparison to others reported in the literature given its short time duration and absence of a simulation element [6,9,10]. Based on the findings in Table 2, this training demonstrated high effectiveness. It is important to note that this study evaluated the self-confidence levels of participants rather than the objective competency of the material. Participants in the post-survey showed more confidence in the knowledge of opioids, identifying an opioid overdose, knowing what steps to take after identification, and knowledge of intranasal naloxone after the training. The effect size for questions evaluating each of these facets of the training was large, as reported by the rank biserial correlation (RBC) in Table 2, indicating a strong efficacy of the training in reaching the objectives. Additionally, the mean±SDs for each of these questions reflect improvement with less deviation, showing a more shared understanding among trainees after the intervention.

When surveyed about prior personal, coursework and clinical experience regarding opioid substance use, post-survey median responses were higher than pre-survey responses with a small effect size. This change may be due to trainees considering our intervention in answering these questions in the post-survey. This reflects the value of the training for some participants personally, academically, and clinically; however, the variability of responses in the post-survey is noted in that every IQR is (1-5) for these questions.

The questions in Table 1 demonstrate the need for training in this cohort by assessing comfort levels of the participants in administering NARCAN® in an emergent situation. The findings here show training is needed to increase comfort levels, as the median response for “even without special training” (2 (1-3)) is lower than that of “only after special training” (4 (3-4)). The questions in Table 3 assess the intervention's impact on the understanding of the administration of naloxone intranasally since there was no simulation element in this intervention. It is well documented in the literature that simulation-based medical training has been shown to be very effective compared to traditional methods of teaching [13]. Despite only utilizing diagrams to simulate emergent naloxone administration, responses reflect understanding of how to act (median (IQR): 5 (4-5)) along with feelings of qualification to take said action (median (IQR): 5 (4-5)) when faced with an opioid overdose. These results reflect efficacy despite not utilizing simulation. Real-life simulation was not included in this training design as it is very time-consuming and requires many additional resources that our team was unable to acquire. These findings highlight a simpler approach that is effective among first-year medical students.

An additional design consideration when creating the Narcan Training Initiative was the length of the training. The intervention featured educational content that had a much shorter duration than programs with similar objectives previously described in literature [6,9,10]. The very inflexible schedule of the preclinical curriculum at our institution made this design imperative. Being able to incorporate this training easily into a preclinical lecture was invaluable to the project. Despite the training being designed differently than others, it still proved useful in meeting the educational objectives determined by our team. This creates an example for medical schools to easily incorporate the Narcan Training Initiative into preset medical activities such as basic life support, Stop the Bleed, active shooter trainings, or didactic lectures. Overall, a training that utilizes less time and resources is easier to incorporate and reproduce.

These findings provide an example to other individuals considering the incorporation of opioid substance use and naloxone administration education into their own communities. It is recommended to utilize local resources to obtain free NARCAN® Nasal Spray to distribute to participants [14]. Future directions for this project include continued improvement of the educational material presented. While the opioid epidemic has been ever-present, it is still changing along with epidemiologic data. This study evaluated perceived confidence rather than objective competency, and long-term knowledge retention was not assessed. Evaluation of long-term retention of the objectives covered by the educational material would also be an appropriate future goal. Lastly, providing this intervention to other audiences would be a meaningful expansion of the analysis. Continuing to evaluate the efficacy of the Narcan Training Initiative while providing meaningful education on life-saving opioid overdose intervention would further address public health issues surrounding the opioid epidemic.

There are some limitations to this study that should be considered. One key limitation is that the outcomes are poorly generalizable to other populations, given that the participants consist of only first-year medical students. While this study centers around targeting preclinical medical students, the training may have limited external validity and may not be as effective when administered to participants of other backgrounds. Given the voluntary nature of the intervention for participants, there is likely response bias as students who completed the entirety of the training may have been more interested in the material. Additionally, the difference in response rates between the pre- and post-survey may have introduced some nonresponse bias to the study. The possibility of this bias was mitigated with the Mann-Whitney U statistical analysis to treat the pre- and post-survey groups independently, in addition to calculating the effect size for each question analyzed. Not taking paired pre-/post-data from this study inhibits the ability to assess long-term retention of this cohort of participants. Lastly, including this intervention in a mandatory education session may have introduced social desirability bias to the participants. Notably, the content on epidemiology of the opioid epidemic included in the presentation may not be relevant to participants outside of the geographic region.

## Conclusions

A brief, student-developed naloxone training delivered within a mandatory preclinical wellness session significantly improved first-year medical students’ self-reported confidence in opioid knowledge, overdose recognition, and intranasal naloxone administration. Despite the absence of a simulation component and the limited duration of the intervention, the large effect sizes observed across primary outcomes demonstrate that concise, resource-efficient educational strategies can meaningfully enhance perceived preparedness to respond to opioid overdose.

This study highlights the feasibility of embedding harm-reduction education into the existing preclinical medical curricular structures without requiring extensive time, personnel, or financial resources. In settings where curricular time is constrained, short didactic interventions may represent a pragmatic alternative to longer simulation-based programs while still achieving substantial educational gains. Furthermore, distributing intranasal naloxone alongside training may reinforce learning and increase the likelihood of real-world application.

While future research should evaluate objective knowledge retention, behavioral outcomes, and long-term impact, these findings support the early integration of overdose response education into undergraduate medical training. Preparing medical students to recognize and respond to opioid overdose aligns with national public health priorities and may contribute to a more responsive, harm-reduction-oriented physician workforce.
